# Near-Infrared Dual Greenhouse Gas Sensor Based on Hollow-Core Photonic Crystal Fiber for Gas-Cell In-Situ Applications

**DOI:** 10.3390/s24051670

**Published:** 2024-03-05

**Authors:** Jianing Wang, Bingqiang Li, Weiping Wu, Guanyu Lin

**Affiliations:** 1 Changchun Institute of Optics, Fine Mechanics and Physics, Chinese Academy of Sciences, Changchun 130033, China; wangjn@ciomp.ac.cn (J.W.); libingqiang@ciomp.ac.cn (B.L.); rainciomp@sina.com (W.W.); 2 University of Chinese Academy of Sciences, Beijing 100049, China

**Keywords:** greenhouse gas, fiber sensor, infrared absorption

## Abstract

A greenhouse gas sensor has been developed to simultaneously detect multiple gas species within a hollow-core photonic bandgap fiber (HC-PBF) structure entirely composed of fibers. To enhance sensitivity, the gas cell consists of HC-PBF enclosed between two single-mode fibers fused with a reflective end surface to double the absorption length. The incorporation of side holes for gas diffusion allows for analysis of the relationship between gas diffusion speed, number of drilled side holes, and energy loss. As the number of drilled holes increases, the response time decreases to less than 3 min at the expense of energy loss. Gas experiments demonstrated detection limits of 0.1 ppm for methane and 2 ppm for carbon dioxide, with an average time of 50 s. In-situ testing conducted in rice fields validates the effectiveness of the developed gas detection system using HC-PBF cells, establishing all-fiber sensors with high sensitivity and rapid response.

## 1. Introduction

The 2020 greenhouse gas bulletin from the World Meteorological Organization emphasizes carbon dioxide and methane as the primary components of greenhouse gases [1]. As per the Kyoto Protocol report, approximately 21% of greenhouse gases arise from agricultural activities [2]. Therefore, the development of a highly sensitive gas detection system tailored explicitly for agricultural applications becomes imperative. Ensuring accurate gas concentration measurements without environmental interference is essential.

Since 1996, HC-PBF has captured researchers’ interest [3] due to its portable and flexible advantages [4,5,6,7]. Highly sensitive species detection, including liquid and gas, is carried out for qualitative and quantitative analysis using different technologies, such as Raman sensing and tunable diode laser absorption spectroscopy [8,9,10]. In gas detection applications, HC-PBFs are commonly used as gas cells. Considering light–gas interaction, HC-PBF maintains at least 95% optical power propagating within its central hollow cores [11]. The primary advantage of HC-PBF lies in its long absorption length despite its compact size, as demonstrated by numerous researchers [12]. However, the prolonged gas filling time into the hollow core hampers HC-PBF sensors from becoming a strong contender in gas sensing [13]. It is crucial to explore and uncover effective methods to enhance the response speed of HC-PBF while evaluating the potential impact of these methods.

HC-PBFs, functioning as gas cells, are commonly linked to single-mode fibers (SMFs) at one or both ends [14,15,16,17]. Using an open end of the HC-PBF represents the simplest approach for gas inlet and outlet [18]. In 2018, L. E. He et al. minimized mode interference effects by employing a free space coupling structure, achieving a methane detection limit of 4.35 ppm. However, due to the single open-ended structure, gas diffusion time extends to nearly 80 min [19]. Employing C-shaped rings spliced to PCF and SMF for connection can create pathways similar to the free space coupling method [17]. Besides gas diffusion driven by concentration gradients, actively exchanging gases can significantly enhance response speed. Vacuum chambers and extreme pressure applications are two common methods for actively increasing gas exchange [20,21]. Valiunas et al. effectively reduced response time, achieving sub-ppmv capability in detecting nitrous oxide gas [20]. However, employing active gas exchange methods inevitably disturbs external gas distribution and may be influenced by mechanical noise. Comparatively, employing multiple drilled side holes in HC-PCF allows for more rapid gas exchange without disturbing environmental gas distribution [22]. Besides the two possible reasons, strand thickness variation and surface roughness scattering, mentioned by T. Frosch, the energy loss from drilled holes should be considered [23]. Nevertheless, the impact of drilled HC-PBF on energy loss and response time requires testing to establish their relationship. Experiment data can provide a balanced trade-off between response time and signal-to-noise ratio.

A greenhouse gas sensor utilizing an all-fiber configuration based on wavelength modulation spectroscopy (WMS) and frequency-division multiplexing (FDM) has been developed for simultaneous multi-species detection. The diffusion gas cell, made from micron-sized diameter HC-PBF, is arranged in a compact setup with multiple side holes drilled into the cell to enhance gas exchange speed. In the experiment, methane and carbon dioxide, as the most typical greenhouse gases, were chosen as representative examples. By optimizing operational parameters, the sensor’s performance, including detection limit, long-term stability, and the relationship among the number of holes, energy loss, and response time, was extensively investigated.

## 2. Materials and Methods

### 2.1. Sensor Configuration

**Figure 1:**
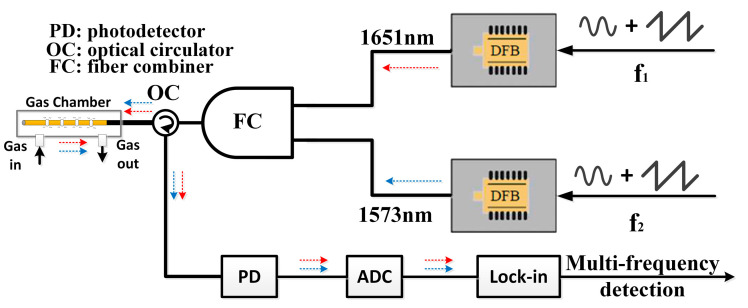
The implemented dual-gas detection sensor designed for in-situ greenhouse applications. PD refers to the photodiode; ADC stands for analog-to-digital converter. The red arrows and blue arrows represent CH_4_ detection channel and CO_2_ detection channel, respectively.

A self-developed dual-gas sensor for methane (CH_4_) and carbon dioxide (CO_2_), based on HC-PBF, has been designed and implemented, with the sensor structure illustrated in Figure 1. The core sensing component is the gas cell formed by HC-PBF and SMFs. The HC-PBF, approximately 0.9 m in length, features an air core of nearly 10 μm, providing an absorption length of over 1.78 m in a reflection structure. Gas exchange occurs through drilled holes in the fiber, facilitating exchange between the interior and exterior gas. At one end of the gas cell, the SMF termination is coated with a reflective surface, reflecting over 95% of the energy back to the input end. The detected signal is then separated by a circulator and directed to the detector.

Two butterfly-packaged 14-pin distributed feedback (DFB) lasers, emitting wavelengths of 1653.7 nm and 1573 nm, are employed to scan absorption lines for methane and carbon dioxide detection, respectively. The gas cell comprises two main parts: an inner HC-PBF-based fiber cell and an outer protection chamber. The original fiber length is one meter. After fusion processing at both ends, the left length is reduced to 0.89 m, which is measured using the time domain reflection method. The laser beam enters the fiber cell through side-drilled holes and undergoes reflection at the opposite side, effectively doubling the absorption length to 1.78 m. The outer chamber serves to mechanically shield and maintain stable working conditions, ensuring consistent pressure and temperature. Analog electrical signals are converged through a sampling and impedance-matching circuit and further processed by a self-developed FPGA circuit. With this all-fiber optical structure, the developed sensor finds application in in-situ greenhouse monitoring, with the potential for optimization towards miniaturization and enhanced portability.

### 2.2. HC-PBF Fiber Characteristic and Fusion

**Figure 2:**
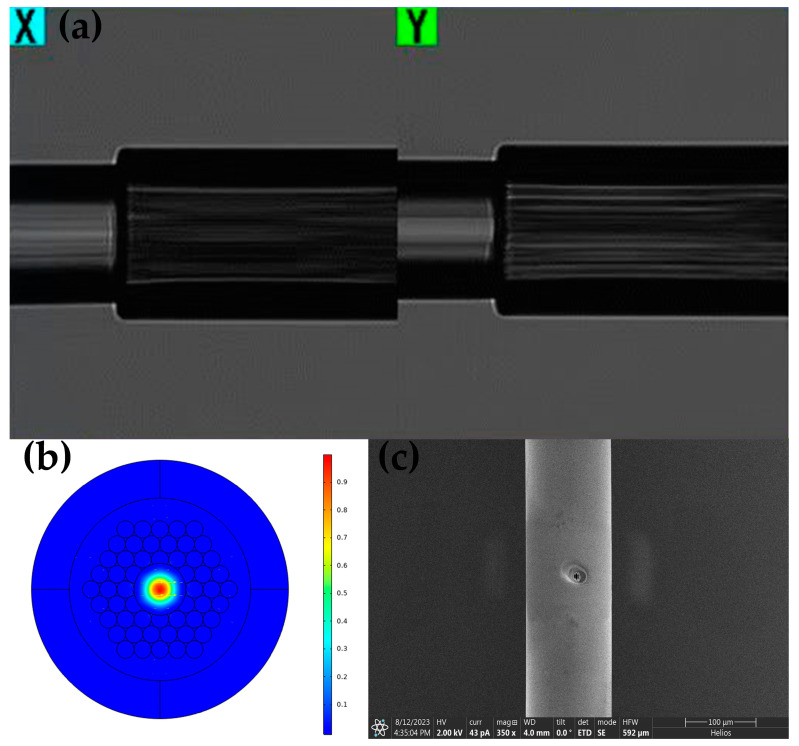
(**a**) SEM image showing the fusion end, (**b**) distribution of the fundamental mode in the HC-PBF, and (**c**) SEM image displaying the surface of the drilled hole.

The HC-PBF utilized in this design, HC-1550-02, is a mature product from NTK. This fiber has an outer diameter of 120 μm, and the central air core measures nearly 10 μm. The bandpass wavelength range is 1490–1680 nm, encompassing the required absorption wavelengths. Within this range, the transmission loss is below 20 dB/km, and the bending radius is approximately 10 cm. The gas cell development involves fusing SMFs with HC-PBF, as depicted in the scanning electron microscope photograph of the fusion joint in Figure 2a. According to finite element analysis (Figure 2b), over 95% of the beam energy is concentrated in the gas-filled hollow-core region, while the remaining energy disperses in the micro-structured cladding region. Energy experiments confirm an energy loss of approximately 0.6–0.8 dB per fusion end face, with fluctuations influenced notably by the process. In the capacity of a gas cell, multiple drilled side holes facilitate gas exchange. These holes have a diameter matching the air core, approximately 10 μm, and are designed as buried holes to mitigate noise and energy loss. The SEM image surface in Figure 2c displays the drilled holes, which measure between 11.2–14.8 μm, slightly larger than the intended size. Experimental energy loss registers between 0.17–0.42 dB with filled nitrogen in fiber, nearly 3.84–9.2% energy loss per hole, indicating a considerable impact of the drilling process on light beam propagation. When the number of drilled holes is increased to seven, the response time is less than 3 min and the energy loss is already higher than 33%.

### 2.3. Dual-Gas Detection Mechanism

**Figure 3:**
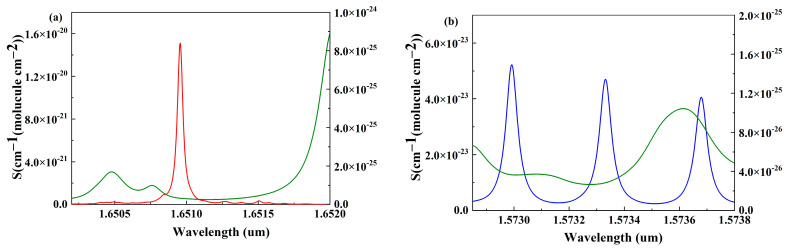
(**a**) Gas absorption spectrum of CH_4_ at 1651 nm, the red and green lines represent CH_4_ and H_2_O absorption lines, respectively; (**b**) gas absorption spectrum of CO_2_ at 1573 nm, the blue and green lines represent CO_2_ and H_2_O absorption lines, respectively.

In consideration of the HC-PBF fiber gas cell application, the absorption lines selected for CH_4_ and CO_2_ should be in the operation wavelength range from 1490 nm to 1680 nm. Therefore, the CH_4_ absorption targeted line selected is around 1650.9 nm which is a typical absorption line for CH_4_ detection. Within this absorption wavelength range, the common interference gases like CO_2_ and H_2_O are at least three times weaker than CH_4_. When the absorption effect of interference gas is very low, this effect is able to be effectively suppressed by signal processing codes based on FPGA. Similarly, the selected absorption lines of CO_2_ are around 1573 nm. The absorption strength ratio between CH_4_ and CO_2_ is nearly 80 which matches approximately the concentration in the atmosphere. In addition, before pumping into the gas chamber, the gas will undergo drying treatment to ensure that the water vapor concentration is not sufficient to affect the detection results. Upon the above design and analysis, the error caused by the interference gas has no effect on the detection precision of CH_4_ and CO_2_ in the selected absorption ranges. Detailed information on selected absorption wavelength ranges is shown in Figure 3.

### 2.4. Gas Cell Structure Design

**Figure 4:**
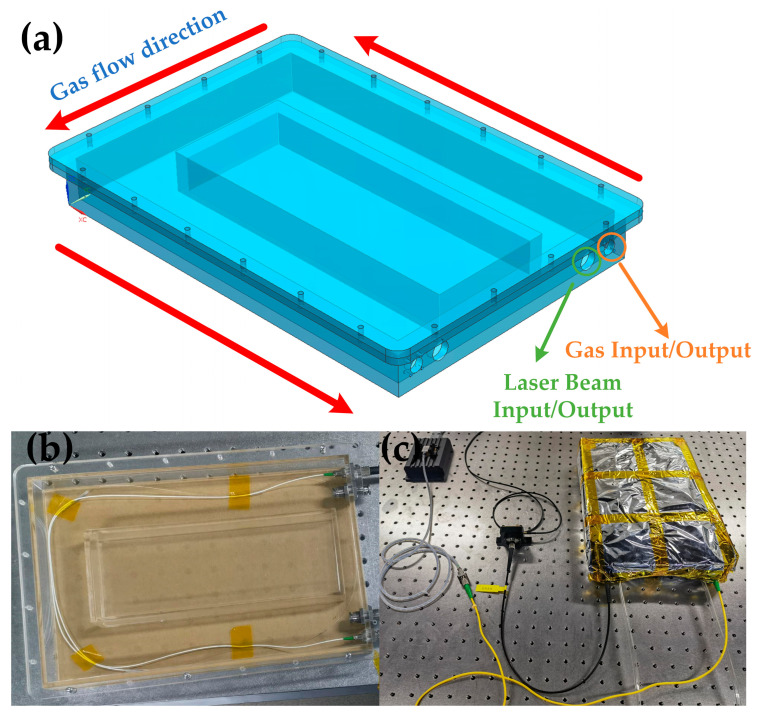
(**a**) Design drawing depicting the mechanical structure of the chamber, (**b**) an image showing the chamber, and (**c**) the gas cell with a thermal package.

The HC-PBF fiber is an exposed fiber with drilled holes. Due to the absence of protective armor, the operational state of the HC-PBF fiber is susceptible to external factors such as vibrations, temperature fluctuations, and airflow. These unpredictable interferences can significantly impact detection results. To mitigate this, a self-designed mechanical protection chamber has been developed and implemented. This chamber serves to shield the HC-PBF fiber from external influences and maintain a stable environmental condition conducive to reliable operation. The mechanical structure of the protection chamber is depicted in Figure 4. The chamber structure is primarily rectangular, with the HC-PBF fiber arranged along the inner wall. Figure 4a shows the design drawing of the mechanical structure, while Figure 4b presents an image of the chamber. The chamber’s central region is filled to minimize internal gas volume, allowing for the pump in of external gases through air vents. Considering the sensitivity of the detection system to environmental temperature fluctuations, thermal insulation is applied to the chamber, as illustrated in Figure 4c.

### 2.5. Harmonic Detection Theory

Wavelength Modulation Spectroscopy (WMS) is commonly employed to enhance the signal-to-noise ratio (SNR) in detection. In the process, a low-frequency periodic saw-tooth signal and a high-frequency sine wave are utilized to scan the absorption lines and modulate the laser’s injection current, respectively. Post modulation, the output light energy from the laser can be expressed as follows:

(1) I(t)=I(t)[1+u(t)+nsin(wt)],

where *u*(*t*) represents the periodic saw-tooth signal, and n is the light intensity modulation parameter. According to the Beer–Lambert law, following the detection of gas absorption, the resulting light energy is

(2) $$I^{\prime }(t)=I_{0}[1+u(t)+\mathrm{nsin}(\omega t)]\exp(-\alpha (t)LC).$$

Therefore, the absorption coefficient can be obtained by using Lorentz linear fitting:

(3) $$\alpha (t)=\frac{\alpha_{0}}{1+(\frac{\upsilon_{t}-\upsilon_{c}}{\upsilon_{FWHM}})},$$

where $\upsilon_{t}$ is the wavelength of the emitted beam with modulation, $\upsilon_{c}$ is the central wavelength of the absorption peak, *a*_0_ is the absorption parameter at the absorption peak, and $\upsilon_{FWHM}$ represents the full width at half maximum. Under typical conditions, the detected gas concentration, which is low, can be expressed as a function of

(4) α[λ(t)]LC<<1,

when the driven output laser wavelength is matched with the absorption peak, the output light signal can be sampled as

(5) $$I^{\prime }(t)=I_{0}[1+\mathrm{nsin}(wt)-\frac{\alpha_{0}LC}{1+m^{2}\sin^{2}(\omega t)}].$$

Following the Fourier series expansion of the aforementioned function, various harmonics can be extracted, including the first harmonic (1*f*) and second harmonic (2*f*). In stable conditions, the output laser energy remains stable, allowing for the establishment of a relationship between the amplitude of the 2*f* signal and gas concentration. Considering environmental influences on the initial laser intensity, the ratio of amplitudes between 2*f* and 1*f* can be employed to mitigate unpredictable variations caused by changes in the initial intensity of the laser. This ratio function can be expressed as

(6) $$\frac{A_{2f}}{A_{1f}}=\frac{-k\alpha_{0}LC}{n},$$

where *k* is the modulation parameter. This design establishes the relationship between $\frac{A_{2f}}{A_{1f}}$ and gas concentration.

In consideration of the previous research, the modulation depth in wavelength modulation should be nearly 2–2.5 times the half width at half maximum of the gas absorption lines. In this case, both the difficulty of hardware utilization of FPGA and the frequency domain distribution of environmental noise are considered. With the signal and noise test, to achieve a satisfying signal and noise ratio, the optimized modulation coefficient is set to 1.9 and 2.1, corresponding to CH_4_ and CO_2_, respectively.

## 3. Results

### 3.1. Waveform Measurements

Compared to previous reports, our research aims to develop an embedded detection system applied for on-situ applications rather than a testing prototype limited to laboratory applications. Therefore, in contrast to employing commercial products, our system is almost entirely self-developed and an implemented detection system, including signal sampling, actuation, and processing. The above-mentioned functions are realized in an embedded system based on an FPGA chip XC7A200T (Xilinx, San Jose, CA, USA), which is a Xilinx Artix-7 type chip. Compared with the detection system using PC, the challenge is how to use the limited hardware logic resources to achieve the expected detection performance. The system clock of this chip is set to 30 MHz with a maximum 120 KHz sample frequency. As a parallel process chip, the synchronization between laser driving and electrical signal demodulation is well ensured. As a price of parallel process, the greatest consumption of embedded logic resources results from iterative operations using multipliers and dividers. In the implementation of all functions, core iterative operations take place in the high-order low-pass finite impulse response (FIR) filter of the digital lock-in amplifier, with its order being directly proportional to the filtering performance. In consideration of the logic source utilization, a cascade integrator comb (CIC) filter and a lower order FIR are used to replace a high order FIR. After trial and error, the CIC order parameter is set to eight with an analog signal sampling of 20 KHz. The order of FIR is set to 246, and the 2*f* signal is extracted every 0.1 s. With a cascade filter, 32,600 logic elements and 110 embedded 18*18 multipliers were set as a standard resource cost to realize the lock-in amplification function.

**Figure 5:**
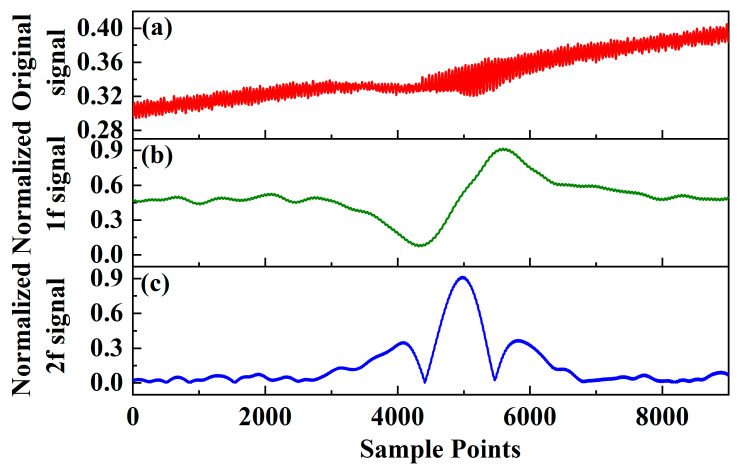
Normalized 2*f* amplitude plotted against modulation depth for optimization purposes. (**a**) the original detecting signal; (**b**) the demodulated 1*f* signal; (**c**) the demodulated 2*f* signal.

To evaluate the sensor’s performance, sample gas mixtures of known concentrations are generated by diluting 500 ppm CH_4_, 2000 ppm CO_2_, and pure N_2_ using a commercial gas dilution instrument. With an absorption length of 1.78 m for the HC-PBF, the waveform is saved and observed using a self-developed CPU based on an FPGA chip. The observed original signal is depicted in Figure 5a. The processed 1*f* and 2*f* signals are displayed in Figure 5b and Figure 5c, respectively.

As a portable sensor, the power consumption of the CPU chip is a significant parameter. When the temperature of the laser is stable, the most power consumption comes from the embedded processor. During this experiment, power consumption was continuously monitored, and the average power consumption was about 1.71 W, including power consumption from the laser, DC-DC chip, FPGA, and other hardware components. It should be noted that power consumption in practical application environments should be re-evaluated because of the unpredictable changes in ambient temperature and variations in the detection period. In this case, the power consumption under the rice field application is lower than 2.12 W. A significant portion of the power consumption is attributed to laser temperature control.

### 3.2. 2f Signal Fitting

The signal-to-noise ratio of the demodulated 2*f* signal, without subsequent algorithm optimization, directly determines the system’s detection performance. The standard deviation, approximately 0.327 mV (1*σ*), was determined within the non-absorption range. There is an inevitable fluctuation in achieving the peak value of the 2*f* signal. To obtain an accurately related 2*f* amplitude signal, the 2*f* signal is fitted before being recorded.

**Figure 6:**
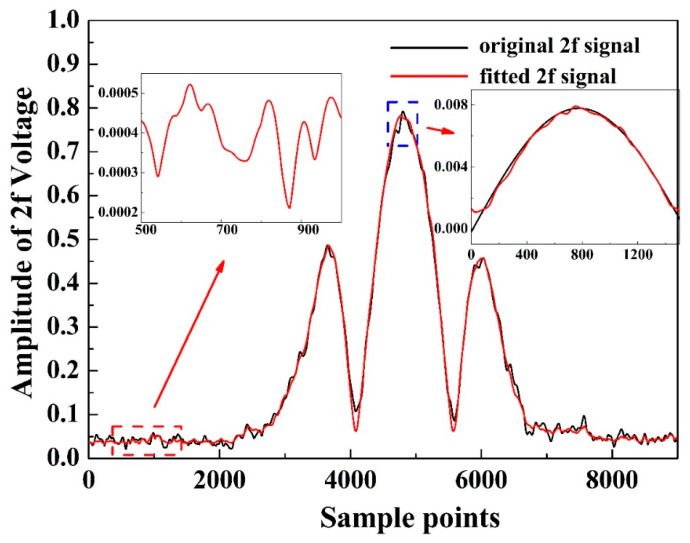
Normalized 2*f* signal flitting and standard deviation.

For comparing hardware resource utilization, the waveform of the 2*f* signal is separately fitted using Gaussian and sine wave fitting techniques. With the same hardware resources, the R-squared values for Gaussian fitting and sine wave fitting are 99.21% and 98.79%, respectively. Figure 6 shows the original demodulated 2*f* signal and the filtered 2*f* signal based on sine wave fitting. The fluctuation around the 2*f* peak signal is effectively suppressed.

### 3.3. Natural Diffusion Process of HC-PCF Cell

**Figure 7:**
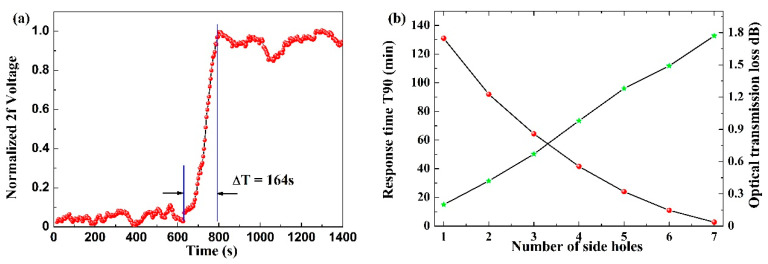
Measurements of the natural diffusion process of CH_4_ into the HC-PCF gas cell by observing the change in the 2*f* signal amplitude. (**a**) Response time with seven drilled holes and (**b**) the impact of the number of drilled holes on response time and energy loss. The red lines and green lines represent the response time and optical transmission loss, respectively.

A specialized experiment was conducted to assess the optimal values for the number of holes and response time. With an increase in the drilled hole count, the corresponding energy loss and response time were recorded. During the experimental process, the protective chamber was purged with pure N_2_ to prevent operational errors stemming from external pressure differences. Dynamic gas distribution was employed instead of static injection distribution for this purpose. In the response experiment, methane was used as the sample, and the amplitude of the 2*f* signal was recorded every second. The results for the response time are depicted in Figure 7a. When the number of drilled holes was increased to seven, the response time decreased to less than 3 min, meeting the requirements for many applications such as precision agriculture and industrial pollution gas detection. Specifically, the decrease in response time exhibited an almost exponential relationship with the number of drilled holes, as illustrated in Figure 7b. Overall, a noticeable reduction in response time resulting from an increased number of drilled holes aligns with some previous findings (e.g., ref. [19]). Discrepancies between our experimental results and reference papers may arise from factors like the total length of the microchannel, the drilled hole placement, and machining errors in their creation. Moreover, for the sake of faster response, each drilled hole consumes approximately 0.2–0.3 dB, implying a trade-off between energy loss and response speed.

### 3.4. Calibration and Data-Fitting

**Figure 8:**
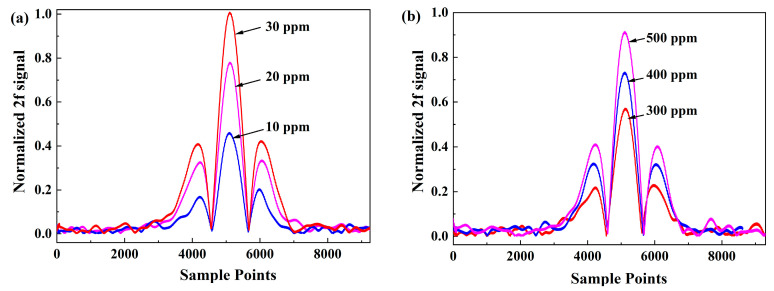
Dot plots for the extracted processed signal with different concentrations of (**a**) CH_4_ and (**b**) CO_2_.

Adjusting to match the gas concentration in the greenhouse, dual-gas calibrations are applied across various concentration ranges. Figure 8a and Figure 8b depicts the representative 2*f* signal amplitudes of the two species at different gas concentrations while maintaining a constant gas pressure, respectively. The data-fitting results demonstrate a linear relationship between 2*f* amplitude and concentration, with R-squared values of 0.996 and 0.993, respectively. The calibrated linear fitting results of CH_4_ and CO_2_ are shown as Equation (7) and Equation (8), respectively. The parameter *C* is the detecting gas concentration, and ∆U is the ratio of 2*f* signal and 1*f* signal.

(7) C=723.94×ΔU−1.121.

(8) C=1214.21×ΔU−17.62.

### 3.5. Detection Limit

**Figure 9:**
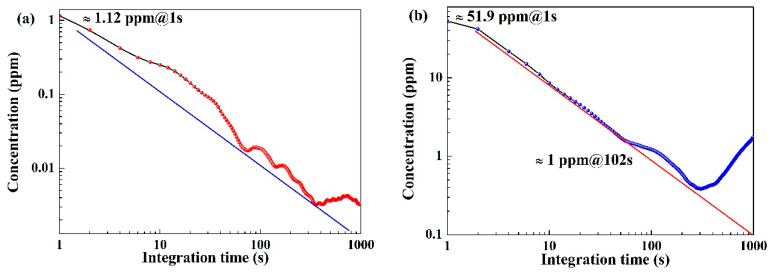
Allan deviation analysis of the sensor system based on one-hour measurements in a pure N_2_ atmosphere concentration of (**a**) CH_4_ and (**b**) CO_2_.

The Allan deviation was employed to determine the minimum detection limit of this system. With an optimal averaging time, the detection system can meet various application requirements across different application backgrounds. In the experiment, pure dry N_2_ was used to flush the gas cell for 180 s before injecting the sample gas. The analysis is based on the Allan deviation. For CH_4_ detection, as depicted in Figure 9a, the 1σ detection limit is approximately 1.12 ppmv for a 1s averaging time. Increasing the averaging time to 50 s improves the detection limit to 0.1 ppm, meeting the detection limit requirement under atmospheric conditions. Due to the weak absorption line, the detection limit for CO_2_ is nearly 51.9 ppm for a 1s averaging time, as shown in Figure 9b. Extending the averaging time to 50 s enhances the detection limit to 2 ppm.

### 3.6. Field Application

**Figure 10:**
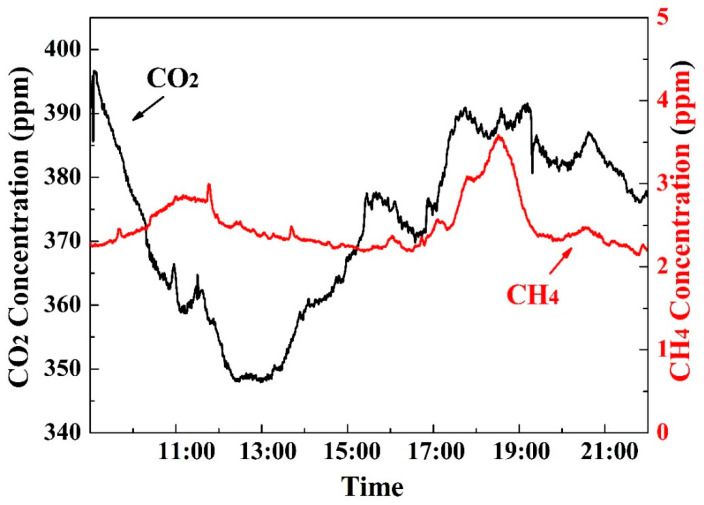
Continuous monitoring of CH_4_ concentration and CO_2_ concentration in a greenhouse on November 2023 in Jilin.

An on-situ test was conducted in a rice field located in Jilin Province, a city in northern China, a typical source and sink for greenhouse gases. Before the on-situ test, a comparative test with a G2401 was carried out to ensure the varying concentrations of water vapor would not influence our test results. Since the experiment took place in October, the selected rice plants were at a mature stage, reaching a height of over 1.2 m. The sampling location was near the roots of the rice field to monitor the greenhouse effect within the group of rice plants. As illustrated in Figure 10, the measured CH_4_ concentration was slightly higher than the average atmospheric concentration, which was marginally lower than the anticipated concentration. The most probable reason is the decreased activity of methanogens in the mature-stage rice plants. Meanwhile, the cultivation method of mature rice is not a flooded mode, which significantly suppresses the generation of CH_4_ from rice fields. In contrast, the concentration of CO_2_ primarily aligned with photosynthesis, indicating satisfactory detection performance. Moreover, the rice field was an outdoor open structure, and the detected gas concentrations were influenced by environmental factors such as wind speed and human activities, contributing to the fluctuations and ripples in the detection results.

Compared to the indoor laboratory environments with nearly constant temperatures without wind, the on-situ application has a more stringent external environmental condition. The most significant influencing factor is the time consumed for temperature stabilization. When the sensor starts up, the time consumed is nearly 90 min, which is 30 times longer than the time consumed in the laboratory. Fortunately, the temperature change in ambient conditions is slow related to the sensor. After the warm-up process, the developed sensor will no longer require any adjustments.

## 4. Discussion

Compared to the other previously reported sensors using a hollow-core photonic crystal fiber, our research has a better performance in gas cell structure design and response time improvement. The single-end reflection structure doubles the absorption length without increasing the sensor volume. The self-developed mechanical protection structure ensures the applicability of the gas cell under in-situ environments, which is verified through testing in rice fields. What is more, the experiment data concerning drilled hole numbers, energy loss, and response time provides a reference for the trade-offs in further design.

## 5. Conclusions

The design and implementation of an in-situ trace gas sensor within an all-fiber structure were presented for the simultaneous detection of multiple greenhouse gas species. Gas experiments were conducted using a gas cell to determine the response time for gas diffusion within the core of an HC-PBF. The experimental results quantified the relationship between the number of drilled holes and response time. However, caution is required in increasing the number of drilled holes due to the associated increase in energy loss. Utilizing the self-developed gas cell, the detection limits for CH_4_ and CO_2_ were determined to be 1.12 ppm and 51.9 ppm, respectively, at 1 s. Performance was notably enhanced to 0.1 ppm and 2 ppm with an average time of 50 s, meeting atmospheric application requirements. An application test in a rice field confirmed the applicability of outdoor use. Future work will focus on improving stability by mitigating the effects of working conditions on the HC-PBF fiber cell. This includes controlling and limiting internal pressure and temperature within a narrower fluctuation range.

## Data Availability

Data are contained within the article.
